# CONTINUOUS PASSIVE MONITORING OF DAILY FUNCTION AND SYMPTOMS IN OLDER ADULTS WITH PROSTATE CANCER

**DOI:** 10.1093/geroni/igae098.2309

**Published:** 2024-12-31

**Authors:** Deanne Tibbitts, Zachary Beattie, Eric Roeland, Rebecca Silbermann, Jason Webb, Gabrielle Meyers, Jeffrey Kaye, Kerri Winters-Stone

**Affiliations:** Oregon Health & Science University, Portland, Oregon, United States; Oregon Health & Science University, Portland, Oregon, United States; Oregon Health & Science University, Portland, Oregon, United States; Oregon Health & Science University, Portland, Oregon, United States; Oregon Health & Science University, Portland, Oregon, United States; Oregon Health & Science University, Portland, Oregon, United States; Oregon Health & Science University, Portland, Oregon, United States; Oregon Health & Science University, Portland, Oregon, United States

## Abstract

Androgen deprivation therapy (ADT) for prostate cancer (PC) increases the risk of physical and cognitive decline as well as side effects impacting quality of life. However, we lack a holistic understanding of the timing and spectrum of ADT-related side effects. Thus, we evaluated the feasibility of using home-based, continuous passive monitoring of daily life functioning, symptom surveys, physical performance, and cognitive testing in patients with PC beginning ADT. We assessed enrollment, passive monitoring uptake, retention, sensor data coverage, and data completion. Passive monitoring included infrared motion sensors (activity), door contact sensors (time out-of-home), watch (activity/sleep), bed mat (sleep), biometric scale (weight), electronic pillbox (medication adherence), and computer-use software (computer habits). We assessed health events and treatment-related symptoms (weekly online survey), physical functioning (short physical performance battery, timed up-and-go test), and cognitive functioning (online cognitive test). We enrolled 17 patients of 52 approached (median age, 73 years); 24 chose not to participate primarily due to concerns of burden (n=10, 42%) and feeling overwhelmed (n=5; 21%). Retention over 14 months was 82% (n=3 withdrawals). Weekly survey completion was 79%. Data coverage for passive monitoring ranged from 55% for watch-based sleep to 98% for motion sensor activity. Testing visit completion across 6 months was 93%; cognitive test completion was 98%. In sum, passive monitoring of daily functioning, symptom surveys, physical performance, and cognitive testing is feasible among older patients with PC beginning ADT. Future research will integrate symptoms, functioning, and health events to examine longitudinal variations in health during ADT.

